# Protein Citrullination: A Proposed Mechanism for Pathology in Traumatic Brain Injury

**DOI:** 10.3389/fneur.2015.00204

**Published:** 2015-09-22

**Authors:** Rachel C. Lazarus, John E. Buonora, Michael N. Flora, James G. Freedy, Gay R. Holstein, Giorgio P. Martinelli, David M. Jacobowitz, Gregory P. Mueller

**Affiliations:** ^1^Program in Neuroscience, Uniformed Services University of the Health Sciences, Bethesda, MD, USA; ^2^US Army Graduate Program in Anesthesia Nursing, Fort Sam Houston, TX, USA; ^3^Department of Anatomy, Physiology, and Genetics, Uniformed Services University of the Health Sciences, Bethesda, MD, USA; ^4^Department of Neurology, Icahn School of Medicine at Mount Sinai, New York, NY, USA; ^5^Center for Neuroscience and Regenerative Medicine, Uniformed Services University of the Health Sciences, Bethesda, MD, USA

**Keywords:** traumatic brain injury, citrullination, astrocytes, calcium, glial fibrillary acidic protein

## Abstract

Protein citrullination is a calcium-driven post-translational modification proposed to play a causative role in the neurodegenerative disorders of Alzheimer’s disease, multiple sclerosis (MS), and prion disease. Citrullination can result in the formation of antigenic epitopes that underlie pathogenic autoimmune responses. This phenomenon, which is best understood in rheumatoid arthritis, may play a role in the chronic dysfunction following traumatic brain injury (TBI). Despite substantial evidence of aberrations in calcium signaling following TBI, there is little understanding of how TBI alters citrullination in the brain. The present investigation addressed this gap by examining the effects of TBI on the distribution of protein citrullination and on the specific cell types involved. Immunofluorescence revealed that controlled cortical impact in rats profoundly up-­regulated protein citrullination in the cerebral cortex, external capsule, and hippocampus. This response was exclusively seen in astrocytes; no such effects were observed on the status of protein citrullination in neurons, oligodendrocytes or microglia. Further, proteomic analyses demonstrated that the effects of TBI on citrullination were confined to a relatively small subset of neural proteins. Proteins most notably affected were those also reported to be citrullinated in other disorders, including prion disease and MS. *In vivo* findings were extended in an *in vitro* model of simulated TBI employing normal human astrocytes. Pharmacologically induced calcium excitotoxicity was shown to activate the citrullination and breakdown of glial fibrillary acidic protein, producing a novel candidate TBI biomarker and potential target for autoimmune recognition. In summary, these findings demonstrate that the effects of TBI on protein citrullination are selective with respect to brain region, cell type, and proteins modified, and may contribute to a role for autoimmune dysfunction in chronic pathology following TBI.

## Introduction

Traumatic brain injury (TBI) is a major cause of injury and death in the US, with over 1.7 million TBIs occurring annually and at least 5.3 million Americans currently living with ongoing disability (1). Traumatic brain injuries in civilians are largely due to automobile accidents as well as falls, sports, and firearms (2). Military populations are at disproportionately elevated risk for blast-related TBI due to the devastating effects of improvised explosive devices (3). While there is a very large body of information on causes and global consequences of TBI, much less is known about the mechanisms underlying long-term pathology.

The long-term consequences of TBI can be complex, and often result in progressive cognitive and behavioral changes. Studies have indicated that anywhere from 10 to 50% of individuals with TBI suffer from persistent symptoms following injury (4), including attention deficits and short-term memory loss (1). This long-term dysfunction follows in the wake of two main injury phases: (1) the primary injury, caused by the immediate forces of the trauma (2, 5); and (2) the subsequent secondary injury, which presents as a constellation of dysfunctional molecular processes including impaired metabolism, free radical production, inflammation, and glutamate excitotoxicity (1). At present, it is not well understood how these various dysfunctional processes following acute injury can lead to progressive, chronic pathology after TBI. This ongoing pathology includes deficits in executive function, attention, processing speed, learning and memory formation and well as behavioral changes in both emotion and affect (1, 4).

A hallmark mechanism of secondary injury following TBI is prolonged imbalance in cellular calcium homeostasis, resulting in excitotoxic calcium overload (6–8). The downstream effects of cellular calcium toxicity have been examined closely in regard to mitochondrial dysfunction and oxidative stress. However, little attention has been given to the role of TBI-induced calcium overload in the activation of peptidylarginine deiminase (PAD) enzymes. This family of calcium-dependent enzymes catalyzes the post-translational modification of citrullination, resulting in the conversion of intrapeptidyl arginine residues to citrulline residues. In addition to altering both the normal structure and function of proteins, citrullination generates “altered-self” epitopes that may be antigenic, prompting autoimmune responses against previously benign proteins (9, 10). Altered calcium homeostasis accompanied by protein citrullination has been implicated in several neurodegenerative disorders, including Alzheimer’s disease (11), temporal lobe epilepsy (12), glaucoma (13), rheumatoid arthritis (14), and multiple sclerosis (MS) (15). In MS, the citrullination of myelin basic protein (MBP) limits the ability of this protein to appropriately associate with lipids (16), which in turn contributes to demyelination by destabilizing sheath structure (17). It has been proposed that the dysfunctional effects of citrullination on myelin sheath structure play a major role in the development of MS (16). Furthermore, citrullinated proteins are also observed within the extracellular plaques seen in post-mortem brains affected by Alzheimer’s disease, suggesting a functional role for this modification in neurodegenerative pathology.

At present, understanding of the specific proteins modified by citrullination is very limited outside the field of immunology, where the antigenic properties of abnormal citrullination are studied largely within the context of rheumatoid arthritis. Additionally, there is no data available regarding the regional and cellular specificity of protein citrullination in neural tissue following TBI. Here, we identify the regions and cell types most susceptible to protein citrullination following TBI, and also identify a subset of neural proteins that are preferentially citrullinated in response to injury. Furthermore, we present the development of an *in vitro* model for simulating TBI through astrocytic calcium excitotoxicity, and identify a citrullinated breakdown product of glial fibrillary acidic protein (GFAP). Until now, citrullination has been primarily associated with chronic, progressive autoimmune and neural disorders. The present findings indicate that protein citrullination is a feature of TBI that may contribute to the long-term pathogenic mechanisms following acute injury, including those that involve the adaptive immune system.

## Materials and Methods

### Controlled cortical impact

Controlled cortical impact (CCI) was conducted as described in Lazarus et al. (18). Briefly, adult male and female Sprague-Dawley rats (Charles River Laboratories, Morrisville, NC, USA) were anesthetized, then subjected to unilateral CCI over the left hemisphere administered through a Impact One stereotaxic impactor (Leica Microsystems, Buffalo Grove, IL, USA), which delivered a 3 mm flat-tipped impactor at 20° to a depth of 2 mm at 5 m/s with a 500 ms dwell time at −3.8 mm bregma in males/−3.0 mm bregma in females. Naïve animals received no anesthesia or CCI treatment. Animals were euthanized 5 days following injury. All animal handling procedures were performed in compliance with guidelines from the National Research Council for the ethical handling of laboratory animals, as approved by the Institutional Animal Care and Use Committee of USUHS (IACUC Protocol APG 12-827, Bethesda, MD, USA).

### Immunohistochemistry

#### Tissue Collection and Preparation

Tissue was collected and prepared as described previously (18). Briefly, euthanized animals underwent transcardial perfusion [phosphate-buffered saline (PBS) followed by 4% paraformaldehyde] after which brains were removed for storage overnight at 4°C in 4% paraformaldehyde and then equilibration in a 30% sucrose solution (2 days, 4°C). Brains were sectioned coronally (20 μm) across the breadth of the lesion site (2.5 mm rostral to 2.5 mm caudal) with a Leica CM1900 cryostat (Leica Microsystems), and sections were then mounted on slides and stored at −80°C. The following groups of gender/conditions were prepared for immunohistochemical analysis: *n* = 11 male rats, CCI; *n* = 8 male rats, naïve control; *n* = 10 female rats, CCI; and *n* = 7 female rats, naïve control.

#### Detection of Cell-Specific Citrullination

The region and cell-specific effects of CCI on protein citrullination were determined using a mouse monoclonal anti-citrulline antibody (mAb 6B3, IgG2b) (19). The antibody was purified from expression medium by Protein A affinity chromatography (HiTrap Protein A HP column (17-0403-01; GE Healthcare, Buckinghamshire, UK) on a GE ÄKTA FPLC fast protein liquid chromatography instrument (FPLC; 18-1900-26; GE Healthcare), aliquoted for single use and stored at −80°C.

Immunodetection of citrullinated proteins was performed as follows. Brain sections were incubated at 4°C overnight with 125 μl of 1:1000 mouse anti-citrulline 6B3 antibody in 0.3% Triton X-100/PBS, with 1:100 normal donkey serum (NDS) as a blocking agent (16 sections/animal). Co-localization experiments were performed by co-incubating these sections with mAb 6B3 and either (1) goat anti-GFAP (1:1000; ab53554; Abcam, Cambridge, England) (astrocytes); (2) goat anti-ionized calcium-binding adapter molecule (Iba1; 1:500; ab5076; Abcam) (microglia); (3) rabbit anti-neuronal nuclei (NeuN; 1:500; ab104225; Abcam) (neurons); or (4) rabbit anti-MBP (1:1000; ab40390; Abcam) (oligodendroglia). Slides were washed five times with 0.2% Triton X-100/PBS and then incubated for 30 min with 125 μl of secondary antibody solution: 1:100 donkey anti-mouse IgG (H + L), conjugated to green-fluorescent Alexa Fluor 488 dye (A-21202; Invitrogen, Waltham, MA, USA); and either 1:100 donkey anti-goat, conjugated to red-fluorescent Alexa Fluor 594 (A-11058; Invitrogen) or 1:100 donkey anti-rabbit, conjugated to red-fluorescent Alexa Fluor 594 (A-21207; Invitrogen). The sections were washed five times with 0.2% Triton X-100/PBS and one time with 1× PBS (5 m) and then visualized with an Olympus BX61 fluorescent motorized system microscope (Olympus, Shinjuku, Tokyo, Japan) using iVision-Mac software (BioVision Technologies, Exton, PA, USA).

#### Preadsorption Control

Specificity of the mAb 6B3 anti-citrulline antibody in immunofluorescence was confirmed through immunoneutralization using a mixture of citrullinated protein molecular weight standards (trypsinogen, glyceraldehyde 3-phosphate dehydrogenase, bovine albumin, trypsin inhibitor, alpha-lactalbumin, carbonic anhydrase, and egg albumin) prepared in-house via 10 h incubation at 37°C with active PAD enzyme cocktail (0.5 μg/μl; P312-37C-25; SignalChem, Richmond, British Columbia, Canada) in Tris buffer (50 mM Tris HCl, pH 7.4); 5 mM CaCl_2_; and 0.73 mM dithiothreitol (DTT) (Sigma-Aldrich, St. Louis, MO, USA). Concurrently, a control sample was prepared in an identical manner, without the addition of the PAD enzyme cocktail.

### Identification of citrullinated protein species in injured rat brain

#### Tissue Collection and Preparation

Brains were collected, snap-frozen with powdered dry ice, and stored at −80°C until use. Brains were thawed on wet ice and then hand dissected to produce blocks of tissue encompassing the lesion site and the surrounding tissue shown by immunohistochemistry to have increased protein citrullination following CCI. The equivalent region from naïve animals (control tissue) was similarly collected. Tissue blocks were homogenized in 5 volumes/tissue weight extraction solution, consisting of: 7.7 M urea; 2.2 M thiourea; and 4.4% CHAPS; also containing 1× complete protease inhibitor mix (Roche). Samples were clarified by centrifugation (20,000 × *g*, 10 min, 4°C), and resulting ­supernatants were fractionated by 2-dimensional electrophoresis (2-DE).

#### Fluid-Phase Isoelectric Focusing

Treatment group pools (control and injured tissue) were prepared (*N* = 4/pool) and fractionated by fluid-phase isoelectric focusing (F-IEF). Briefly, 200 μl aliquots of each pool were diluted up to 2.865 ml in IEF running solution (7.7 M urea; 2.2 M thiourea; 4.4% CHAPS; ampholytes (150 μl, pH 3–10; ZM0021, Invitrogen); DTT (50 μl, 2 M); and bromophenol blue (10 μl, 10 mg/ml). Samples were loaded into the ZOOM IEF Fractionator (ZMF10002; Invitrogen) and focused according to the following conditions: (1) 100 V, 1.2 mA, 0 W (15 min); (2) 200 V, 2.0 mA, 0 W (1 h); (3) 400 V, 2.0 mA, 1 W (1 h); and (4) 600 V, 1.5 mA, 1 W (1 h) resulting in fractions of proteins within the following five pI ranges: 3.0–4.6; 4.6–5.4; 5.4–6.2; 6.2–7.0; and 7.0–9.1.

#### Molecular Weight Fractionation

Proteins in each of the F-IEF fractionations were further resolved by molecular weight fractionation using conventional one-dimensional gel electrophoresis. Samples were combined with an equal volume of 4× reducing loading buffer (Novex NuPAGE LDS sample buffer; 50 mM DTT; Invitrogen), heated at 70°C (20 min) and then fractionated (10 μl/well) using NuPAGE 4–12% Bis–Tris gels (Novex, Invitrogen), using 1× MES (2-[*N*-morpholino]ethanesulfonic acid) running buffer (Novex, Invitrogen). Proteins were transferred to nitrocellulose blots using an iBlot transfer apparatus and gel transfer stacks (Nitrocellulose Mini; 1B301002, Invitrogen).

#### Immunoblotting

Blots were blocked with 5% instant non-fat dry milk/Tris-buffered saline/Tween 20 (TBS-T) (1 h, room temperature) and then incubated with the 6B3 primary antibody (1:300 in TBS-T; mAb stock = 1.79 mg/ml) for 1 h at room temperature, then 4°C overnight. Following equilibration to room temperature (30 min), membranes were washed in TBS-T (five times over 60 min), incubated with secondary antibody, horseradish peroxidase-labeled, goat anti-mouse IgG (1:2500 in 5% TBS-T; 31430, Thermo Scientific) for 2 h at room temperature and then visualized by enhanced chemiluminescence (ECL) (Novex ECL HRP Chemiluminescent Substrate Reagent Kit; WP20005, Invitrogen) using the FUJI LAS 3000 Imager (Fujifilm, Minato, Tokyo, Japan). Images collected were analyzed using MultiGauge software (v. 3.0, Fujifilm). In some cases, blots were reprobed with a second anti-protein citrulline antibody (1:500; MABN328EMD; Millipore; detection with horseradish peroxidase-labeled, goat anti-mouse IgM; 1:2500 in TBS-T; 31440, Thermo Scientific), with final overnight washing, to confirm the 6B3 immunoreactive features and increase the sensitivity of detection. No new signals were revealed by this approach.

The specificity of mAb 6B3 for detecting citrullinated proteins on western blots was confirmed by immunoneutralization, similar to the approach used for immunohistochemistry (see above), but involving solid-phase preadsorption (versus fluid phase) to accurately replicate the conditions of western blotting. In this case, the 6B3 antibody was reacted with a strip of nitrocellulose (Protran^®^ BA85, 0.45 μm pore size, binding capacity 80 μg/cm^2^; Sigma-Aldrich; cut into 1.4 cm × 3 cm pieces) to which either the citrullinated or native forms of fibrinogen had been bound. The amount of protein absorbed to the strip was 200 μl/200 μg of citrullinated human fibrinogen (400076, Cayman Chemical, Ann Arbor, MI, USA), or 200 μl/200 μg human fibrinogen (16088, Cayman Chemical) in TBS-T. The duration of the antibody absorption was 16 h at 4°C.

#### Protein Identifications

Immunoreactive signals of interest were mapped to corresponding features in Coomassie-stained gels. These features were excised and processed for identification through the ESMS-Basic Protein ID service using an Elite Orbitrap mass spectrometer (Thermo Scientific) by the W. M. Keck Mass Spectrometry and Proteomics Resource (W. M. Keck Foundation Biotechnology Resource Laboratory, New Haven, CT, USA). Analyses results were received through and tracked within the Yale Protein Expression Database (YPED) (Yale/NIDA Neuroproteomics Center, New Haven, CT, USA). Criteria for a positive identification included an expectation score <1.0E−65, a percent coverage >35.0%, and a pI and mw that were consistent with the fractionation data.

### *In vitro* model of protein citrullination following TBI-induced excitotoxicity in astrocytes

An *in vitro* model of simulated TBI was established using normal human astrocytes (NHA CC-2565, Lonza, Allendale, NJ, USA) and treatment with the calcium ionophore, ionomycin (Free Acid, Streptomyces conglobatus, 407950, Calbiochem, Billerica, MA, USA), to simulate calcium excitotoxicity. Astrocytes were cultured to ~70% confluence in T-75 tissue culture flasks according to vendor instructions. Cells were washed with TBS (four times) and then treated for 4 h (37°C, 95% O_2_/5% CO_2_) with either ionomycin (10 μM; 10 ml TBS) or dimethyl sulfoxide vehicle (DMSO; 10 μl/10 ml TBS). Following incubation, protease inhibitors were added to the flasks (final concentration of 1×; Complete protease inhibitor mix; 10269700, Roche) and cells were harvested by scraping and centrifugation (800 × *g*, 4°C, 10 min). Cells were extracted by vortexing into IEF denaturant (see Fluid-Phase Isoelectric Focusing) at a ratio of 1 part pellet to 4 parts buffer (v/v). Samples were clarified by centrifugation (15,000 × *g*, 4°C, 10 m). The resulting supernatant was processed by one-dimensional gel electrophoresis and immunoblotting as described above. Replicate immunoblots containing samples from three separate experiments each were probed with either anti-citrullinated protein (6B3) or anti-GFAP (1:3000, Z0334, DAKO, Carpinteria, CA, USA). Each primary antibody was paired with an appropriate secondary antibody (6B3: horseradish peroxidase-labeled, goat anti-mouse IgG; 1:2500; 31430; Thermo Scientific; GFAP: horseradish peroxidase-labeled, goat anti-rabbit IgG; 1:3000; HAF008; R&D Systems) and blots were visualized by ECL.

### Statistical analysis

All animals in the study were scored using four brain sections per animal. Citrullination immunohistochemistry was quantified in the cortex using a previously developed scoring system (18), with a score of 0 reflecting the lowest amount of citrullination fluorescence intensity in a region and a score of 3 reflecting the greatest amount of fluorescence intensity (see Figure S1 in Supplementary Material). Four brain sections per animal were scored, and comparisons between groups were analyzed by ANOVA with a Tukey HSD *post hoc* test (IBM SPSS Statistics for Macintosh, Version 22.0, Armonk, NY, USA: IBM Corp.). A value of *p* < 0.05 was considered significant.

## Results

**Figure 1:**
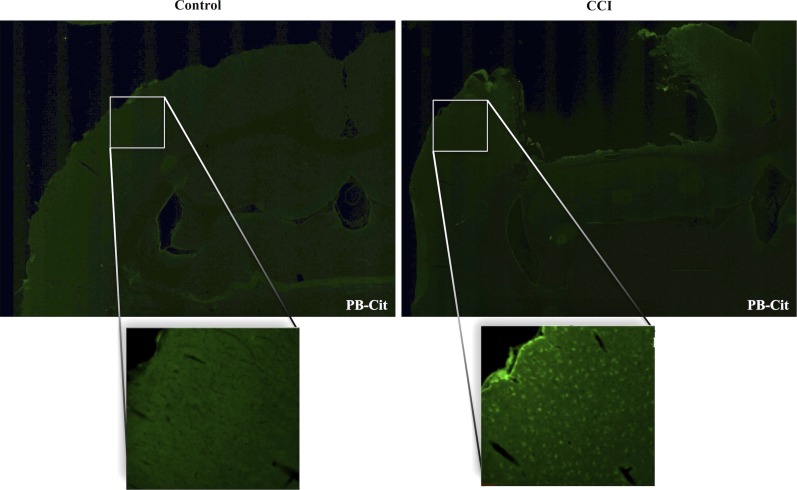
**Injury upregulates the expression of citrullinated proteins in the cerebral cortex**. Brain tissue was collected 5 days after CCI and evaluated for protein citrullination by anti-protein citrulline immunolabeling using mAB 6B3. The upper panels show immunolabeling in sections of control (left) and injured brain (right) (2× magnification). Lower panels show higher magnifications of cerebral cortex adjacent to the lesion site (right) or a comparable site in control cortex (left) (20× magnification). Data are representative of 15 control animals (8 males and 7 females) and 21 CCI animals (11 males and 10 females). No gender-based differences were observed. PB-cit, protein-bound citrulline.

Figure 1 presents the effects of CCI on the expression of citrullinated proteins in the rat cerebral cortex. Immunohistochemical analysis demonstrated that the basal level of protein citrullination as detected by labeling with mAB 6B3 was very low under control conditions. In contrast, CCI induced a marked increase in the immunolabeling of cells in the cortex. This up-regulation of protein citrullination was most pronounced in the vicinity of the lesion. The morphology of the affected cells was remarkably consistent, suggesting that the effects of CCI on protein citrullination were cell specific.

**Figure 2:**
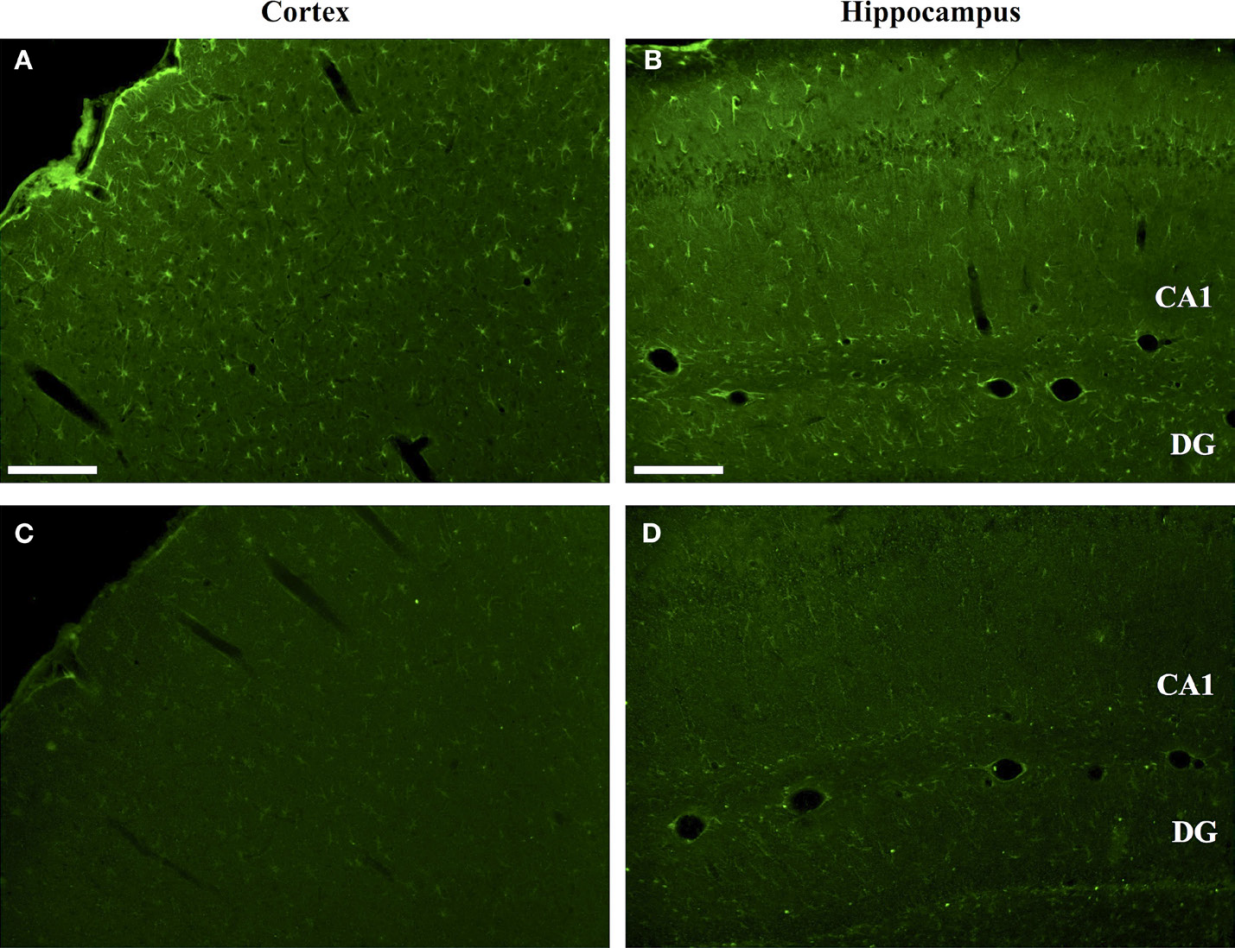
**Specificity of anti-protein citrulline immunolabeling by mAb 6B3**. **(A,B)** show immunofluorescent signals from mAb 6B3 anti-protein citrulline labeling of the injured cerebral cortex **(A)** and ipsilateral hippocampus **(B)**. **(C,D)** shows immunolabeling of equivalent sections with mAb 6B3 that was preadsorbed with citrullinated protein standards. The control and immunoneutralized preparations of mAb 6B3 were treated identically, with the exception of the presence or absence of neutralizing citrullinated proteins. Data are representative of two independent experiments. Scale bar: 200 μm.

The specificity of mAb 6B3 labeling of citrullinated proteins in TBI brain was confirmed by immunoneutralization. As shown in Figure 2, preabsorption of the mAb 6B3 with citrullinated protein standard effectively eliminated the labeling of cells in the cerebral cortex and hippocampus of CCI brain. In this experiment, the active labeling condition utilized mAb 6B3 that had been exposed to the identical absorption procedures as the neutralized mAb 6B3, except that the neutralizing proteins were not citrullinated under the control condition. Additionally, a secondary-only control was also conducted and further confirmed the specificity of the anti-citrullinated protein labeling (not shown).

**Figure 3:**
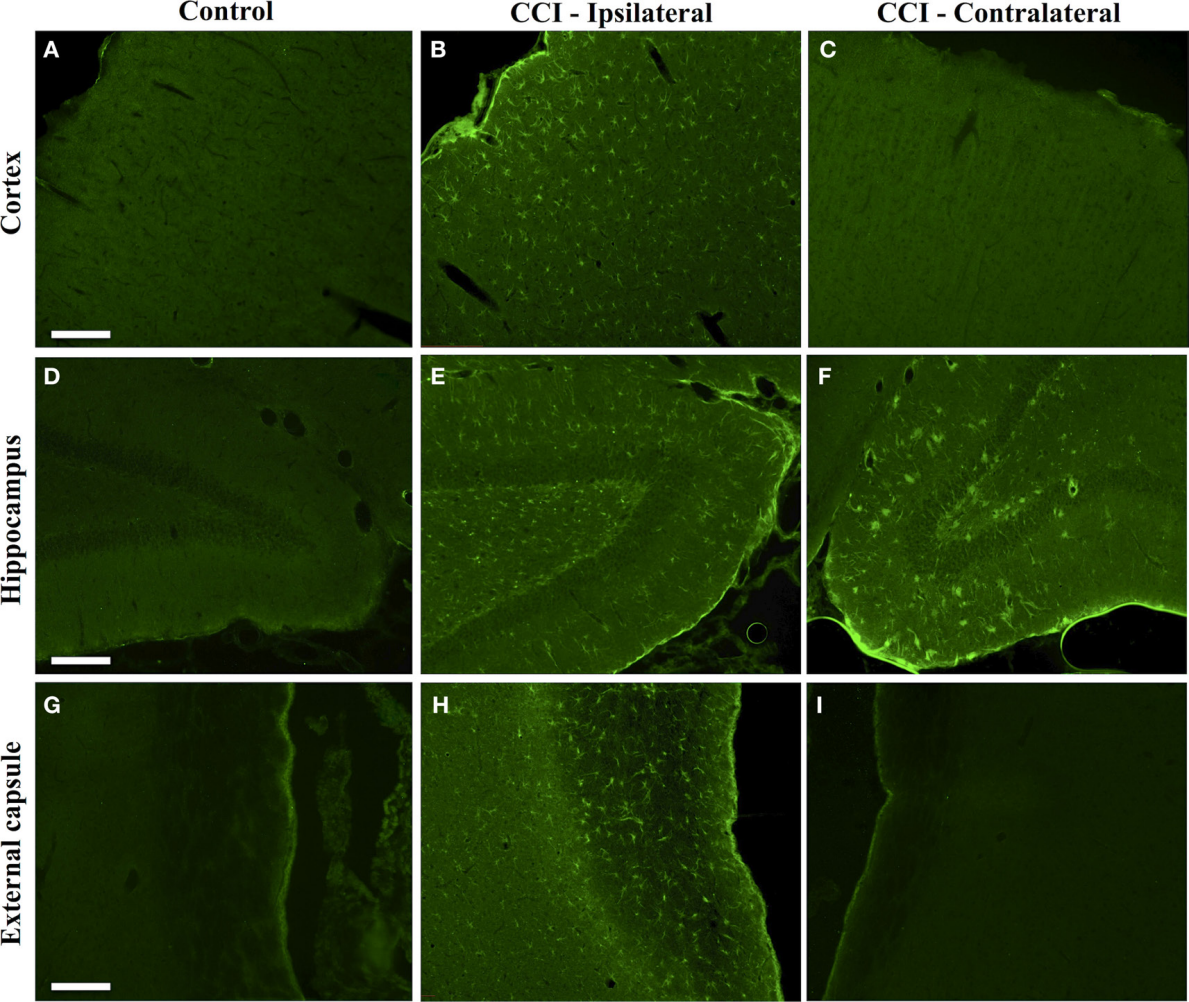
**Increased protein citrullination in the cerebral cortex, hippocampus and external capsule following CCI**. Anti-protein citrulline immunolabeling by mAb 6B3 is shown for the control brain regions **(A,D,G)**, regions ipsilateral to the lesion **(B,E,H)**, and regions contralateral to the lesion **(C,F,I)**. Structures represented are the cerebral cortex **(A–C)**, hippocampus **(D–F)**, and external capsule **(G–I)**. Data are representative of 15 control animals (8 males and 7 females) and 21 CCI animals (11 males and 10 females). No gender-based differences were observed. Scale bar: 200 μm.

The regional effects of CCI on protein citrullination are summarized in Figure 3 and Figure S2 in Supplementary Material. CCI produced a marked increase in protein citrullination throughout the injured cortex, extending from lateral to the lesion site to regions of the cortex not directly impacted by CCI (Figure 3B). Scoring of this region revealed that male naïve rats (mean score: 0.06) and male CCI rats (mean score: 1.43) were significantly different, *p* < 0.001, and female naïve rats (mean score: 0.04) and female CCI rats (mean score: 1.35) were also significantly different, *p* < 0.001 (*n* = 11 male rats, CCI; *n* = 8 male rats, naïve control; *n* = 10 female rats, CCI; *n* = 7 female rats, naïve control) [*F*(3,32) = 31.05, *p* = 0.000, with *post hoc* Tukey HSD analyses considering injury and gender variables independently]. Similarly, immunolabeling of the injured ipsilateral hippocampal formation (Figure 3E) and external capsule (Figure 3H) showed similar increases in protein citrullination in these regions. To a lesser extent, protein citrullination was also observed in fibers extending ventrally from the lesion site toward the midline corpus callosum (not shown). Other brain regions, including the amygdala and caudatoputamen, were completely negative for protein citrullination in these CCI animals. Furthermore, scoring of the cortex revealed that there was no gender difference observed in the regionalization or magnitude of protein citrullination following injury (CCI: *p* = 0.968; naïve: *p* = 0.999). In general, CCI appeared to have little effect on the status of protein citrullination in contralateral brain structures, with the exception of the dorsal hippocampus, where ~20% of injured animals (4 of 21) displayed an intense labeling of unusually large and rounded cells (Figure 3F). Finally, 6B3 immunolabeling was uniformly low across all regions studied in control male and female animals (Figures 3A,D,G).

**Figure 4:**
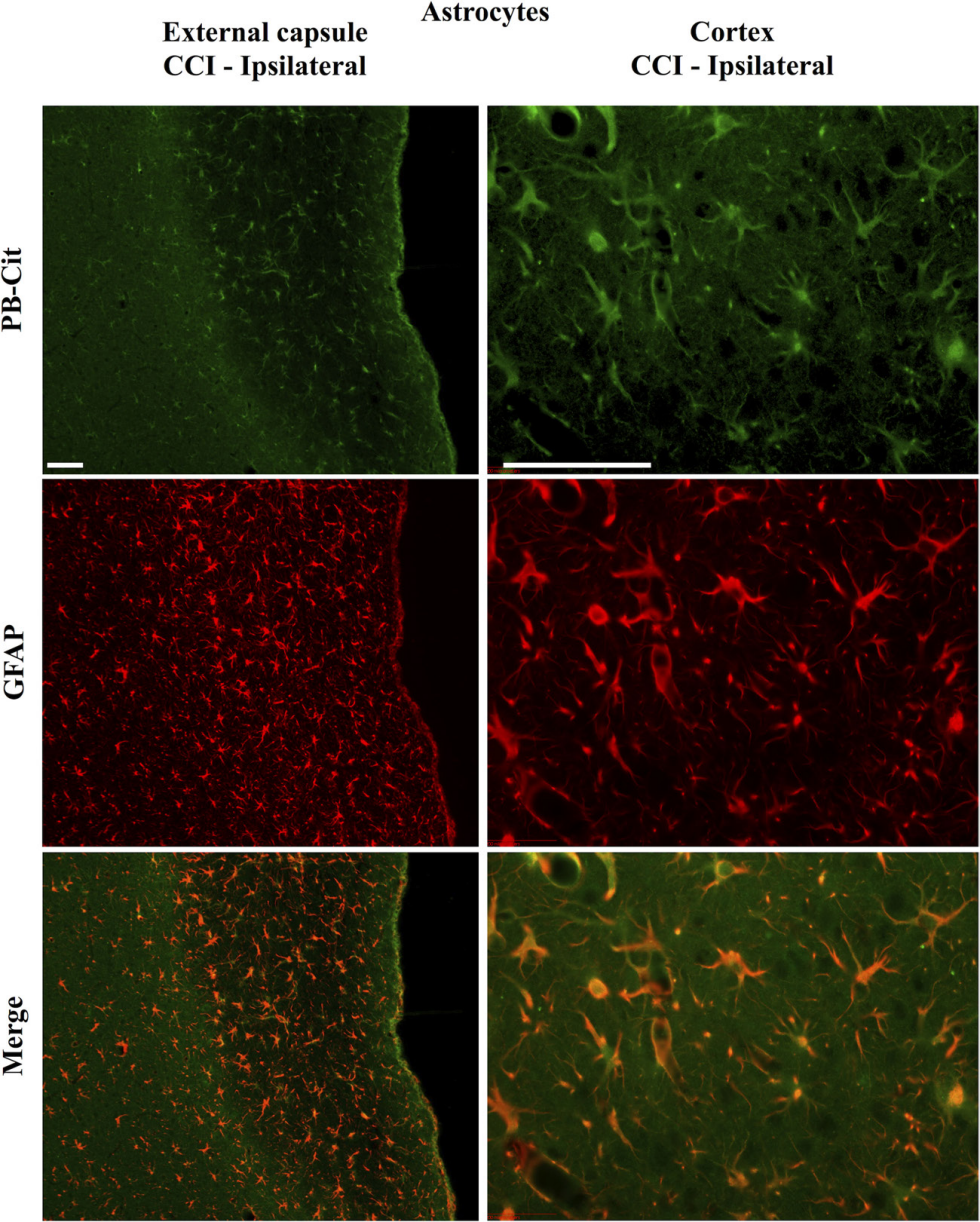
**Localization of CCI-induced protein citrullination to astrocytes**. Panels on the left show the co-localization of mAb 6B3 labeling with anti-GFAP labeling in the external capsule. Panels on the right show the co-localization of mAb 6B3 labeling with anti-GFAP labeling in the cerebral cortex. Data are representative of 15 control animals (8 males and 7 females) and 21 CCI animals (11 males and 10 females). No gender-based differences were observed. Scale bar: 200 μm. PB-cit, protein-bound citrulline.

**Figure 5:**
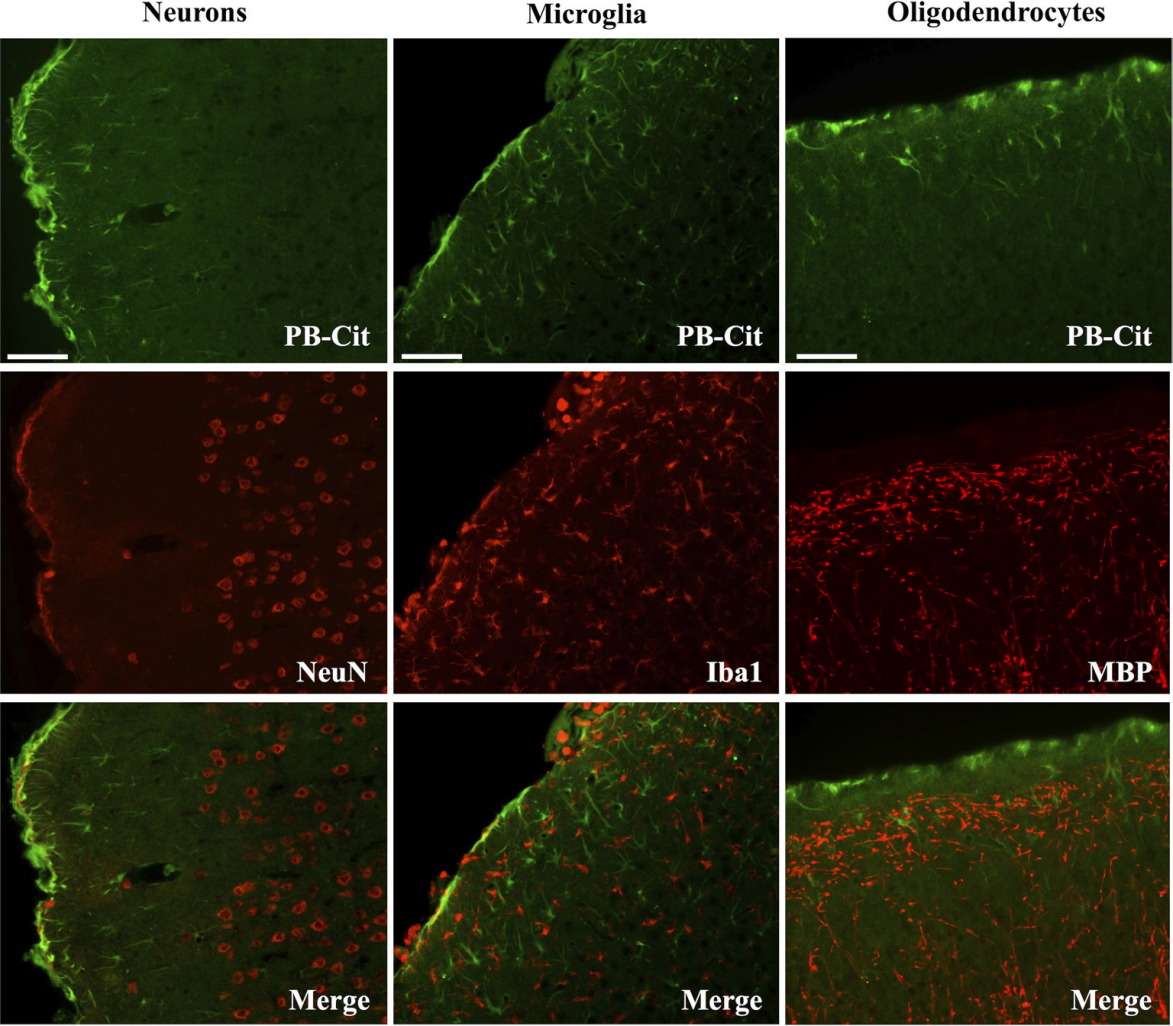
**CCI did not affect the status of protein citrullination in neurons, microglia or oligodendrocytes**. Sections of cerebral cortex ipsilateral to CCI were probed with mAb 6B3 to label protein-bound citrulline (PB-cit) (upper panels) and either anti-NeuN, Iba1, or MBP to label neurons, microglia, or oligodendrocytes, respectively (middle panels). The merge of the two signals is presented in the lower panels. Data are representative of eight separate experiments. Scale bar = 200 μm.

**Figure 6:**
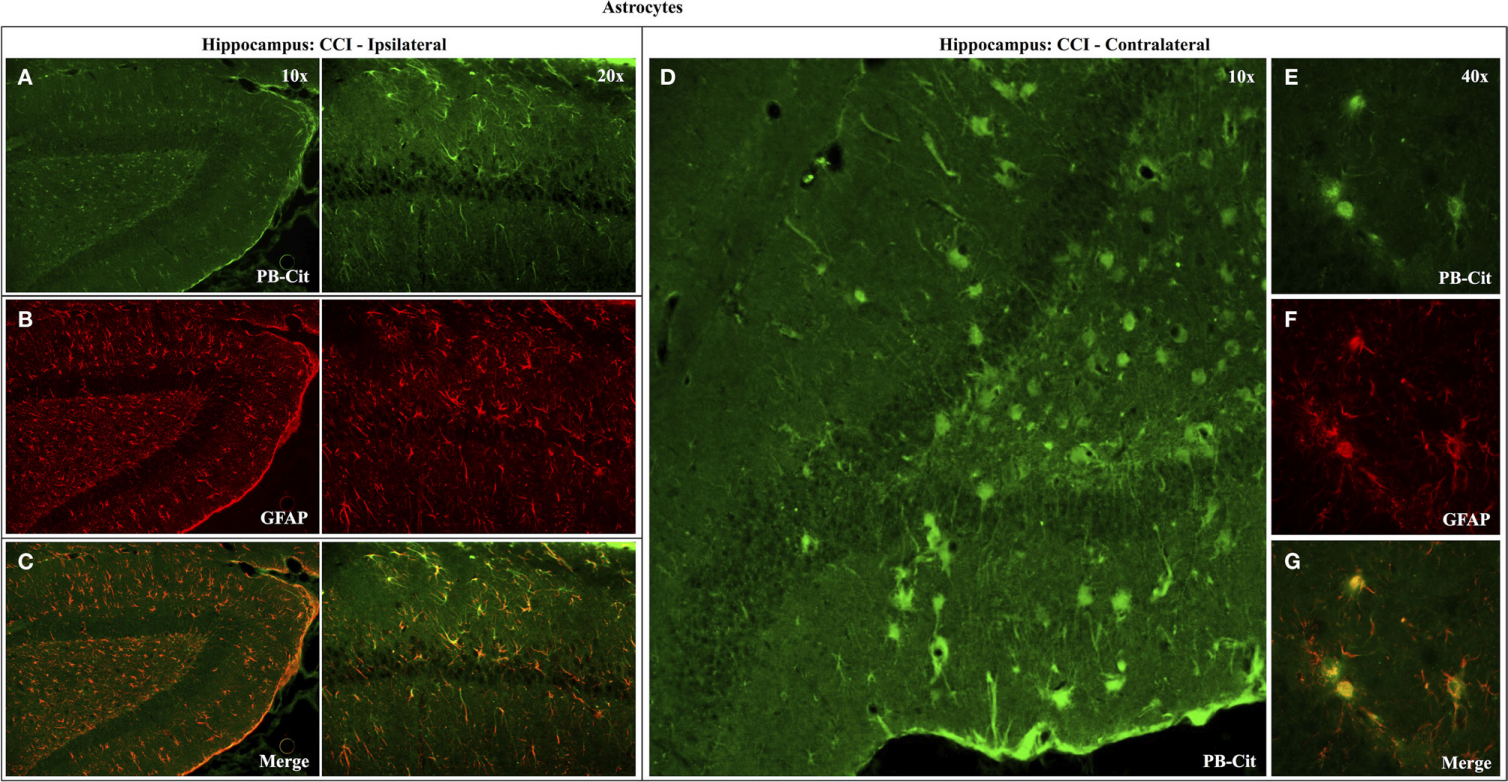
**Effects of CCI on protein citrullination in GFAP-positive cells of the ipsilateral and contralateral hippocampus**. Co-localization of anti-protein citrulline and anti-GFAP labeling in astrocytes of the ipsilateral hippocampus is depicted in the pairs of panels presented on the left **(A–C)**. **(D)** Shows the distinctive anti-citrullinated protein immunolabeling of large, rounded cells observed in the contralateral hippocampus of ~20% of CCI animals (4 of 21). While these cells lacked the classical stellate morphology of astrocytes, the anti-citrullinated protein labeling co-localized with GFAP immunolabeling **(E–G)**. PB-cit, protein-bound citrulline.

Dual immunofluorescence revealed astrocytes to be the principal cell type in which protein citrullination was affected by CCI. Figure 4 shows that anti-citrulline labeling in the cortex and external capsule was predominantly co-localized with GFAP. Similar observations were made in other affected brain regions. The findings presented in Figure 5 further confirm that CCI-induced protein citrullination was not significantly associated with neurons (NeuN), microglia/macrophages (Iba1), or oligodendrocytes (MBP) in the cortex. Citrullination was also not significantly associated with these cell types in any other brain regions investigated (not shown). It should be noted that the lack of co-localization of protein citrullination with NeuN immunoreactivity might reflect diminished NeuN expression following TBI, rather than a lack of citrullinated proteins within neurons. The distinctive profile of CCI-induced protein citrullination in the ipsilateral and contralateral hippocampus is shown in Figure 6. As noted above, increased anti-citrullinated protein labeling was clearly evident in the ipsilateral hippocampus of all animals studied (Figure 6A), and this labeling co-localized with GFAP labeling (Figures 6B,C). In addition, in ~20% of injured animals, the contralateral hippocampus also displayed intense 6B3 labeling (Figure 6D) that co-localized with GFAP labeling. The co-labeled cells were morphologically distinct from traditional stellate astrocytes, displaying a rounded, branchless appearance consistent with that of a macrophage (Figures 6E–G).

**Figure 7:**
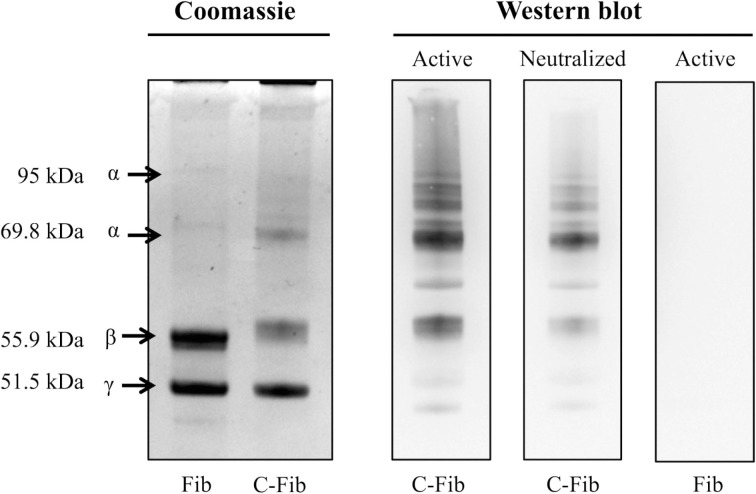
**Specificity of anti-protein citrulline mAb 6B3 detection for western blotting**. Displayed on the left are two Coomassie-stained profiles showing the protein composition of native fibrinogen (Fib) and citrullinated fibrinogen (C-Fib). The western blot (right) shows three immunoblots in which citrullinated fibrinogen was probed with active mAb 6B3 (Active/C-Fib; left lane); citrullinated fibrinogen was probed with immunoneutralized mAb 6B3 (Neutralized/C-Fib; middle lane); and native fibrinogen was probed with active mAb 6B3 (Active/Fib; right lane).

Figure 7 demonstrates the specificity of the 6B3 antibody for detecting citrullinated proteins in a western blot format. Shown on the left is the Coomassie staining for human fibrinogen (Fib) and the same preparation of fibrinogen that was enzymatically citrullinated by reaction with PAD4 (C-Fib). The protein staining shows that the characteristic profile of purified human fibrinogen is modestly affected by reaction with PAD4. Presented on the right are immunoblots showing the reactivity of mAb 6B3 with the citrullinated fibrinogen preparation (Active/C-Fib) and the elimination of this reactivity by preabsorption of 6B3 with citrullinated fibrinogen prior to blotting (Neutralized/C-Fib). There was no reactivity of mAb 6B3 with fibrinogen that had not been citrullinated by PAD4 treatment (Active/Fib).

**Figure 8:**
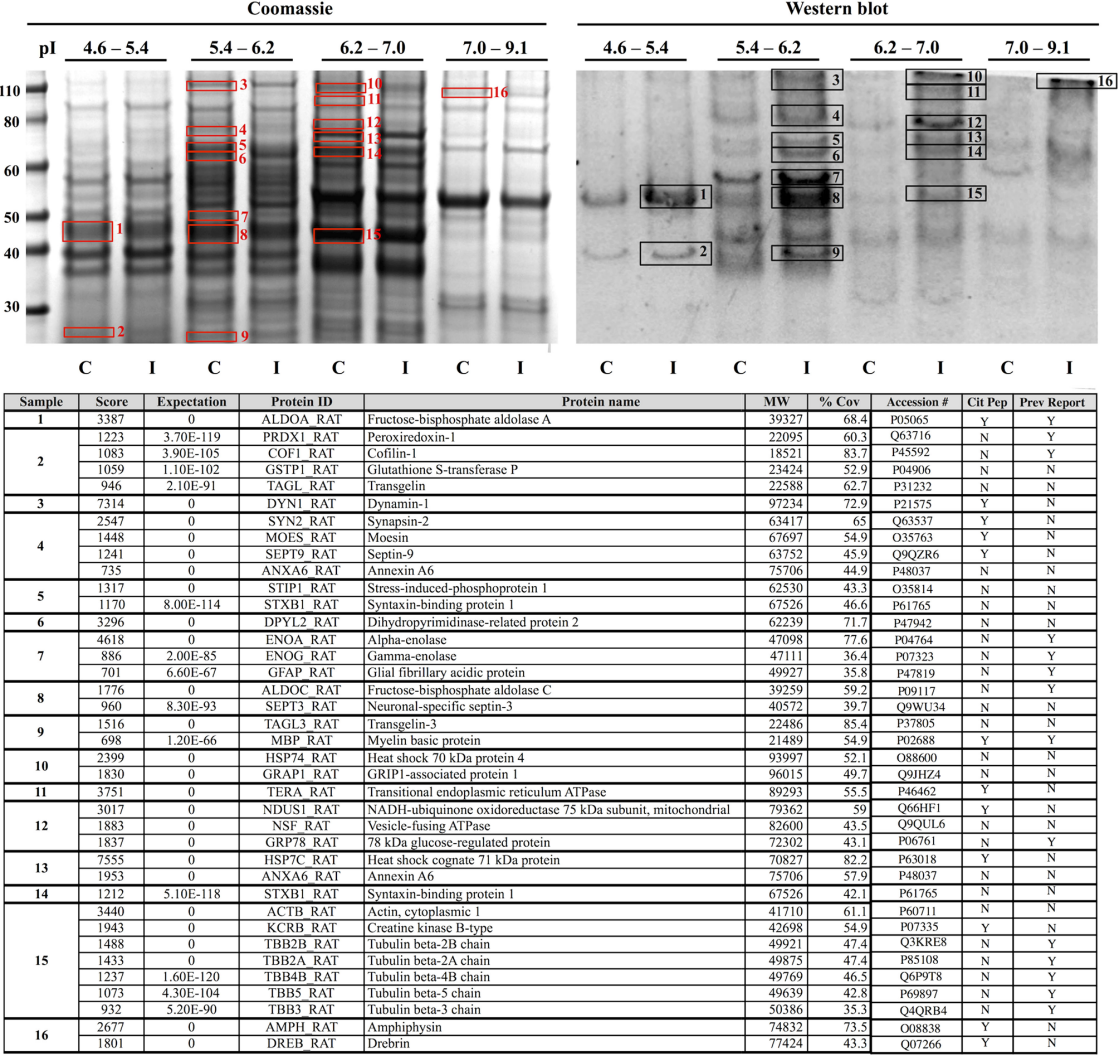
**Identification of proteins that are citrullinated in response to CCI**. Extracts of control (C) and injured (I) cerebral cortex were fractionated by fluid-phase isoelectric focusing into defined pH ranges (shown at top) and then further resolved according to molecular weight using one-dimensional gel electrophoresis. Proteins were then transferred to nitrocellulose membranes and probed for protein-bound citrulline (see “Materials and Methods”) (right panel, “Western blot”). Gels run in parallel were visualized with Coomassie (left panel). Sixteen features showing increased citrullination in response to CCI (black numbered boxes, right panel) were mapped to corresponding Coomassie features (red numbered boxes, left panel) and identified by peptide mass finger printing and tandem mass spectrometry. Proteins identified are listed in the lower panel. Analysis of the mass spectra data set with a variable modification search for the citrullination of arginine residues revealed several proteins with sites of citrullination on arginine residues with Mascot score corresponding to *p* < 0.05 (Cit Pep). Several of these proteins (Prev Report) are also citrullinated in other pathologies, including ALDOA, ALDOC, GFAP, PRDX1, COF1, ENOA, ENOG, MBP, and tubulin (beta) in prion disease (20, 21); GFAP and MBP in Alzheimer’s disease and multiple sclerosis (16); ENOA in rheumatoid arthritis (22); and GRP78 in Type I diabetes (23). Images are representative of six independent experiments. A total of *n* = 4 CCI and *n* = 4 control animals were examined.

Proteomic analysis revealed that the effects of CCI on protein citrullination were specific to a discrete subset of proteins making up the entire brain proteome, and further, that the proteins involved are primarily associated with cytoskeletal structure and metabolic processes (Figure 8). Shown in the upper left panel are the proteomes of control and injured cerebral cortex fractionated by fluid-phase isoelectric focusing. Each pair of lanes, control (C) and CCI (I), show the proteins present in the four different pI partitions. As visualized by Commassie staining, CCI did not appreciably affect the general pattern of protein staining across the four pI fractions. In contrast, the pattern of protein citrullination was dramatically impacted by CCI (upper right panel). Consistent with immunohistochemistry findings (Figures 2 and 3), little protein citrullination was observed in control cortex (C), whereas CCI (I) resulted in the intense labeling of a distinctive subset of the fractionated proteins. The immunoreactive signals of the western blot were mapped to Commassie features of the protein gel, and proteins were identified by peptide mass fingerprinting and tandem mass spectrometry. The proteins identified are presented in the lower panel of Figure 8. These proteins are functionally grouped as cytoskeletal components (including dynamin-1, GFAP, and several forms of tubulin); those involved in metabolic processes (including peroxiredoxin-1, dihydropyrimidinase-related protein 2, and creatine kinase B-type); and proteins involved in cell–cell signaling and synaptic transmission (synapsin-2, syntaxin-binding protein 1, and amphyiphysin). It should be noted that these functional groups of affected proteins – cytoskeletal components, metabolic proteins, and proteins involved in cell–cell signaling – may have been preferentially identified as citrullinated due to their relatively high abundance. These abundant protein types may be more easily identifiable through proteomic analyses than less abundant proteins, which may also be citrullinated but not easily identified. In this regard, however, only a small proportion of the very high abundance proteins were identified as being citrullinated, indicating a notable degree of specificity in the citrullination response.

Analysis of the mass spectra data set with a variable modification search for the citrullination of arginine residues revealed many sites of citrullination on arginine residues with Mascot score corresponding to *p* < 0.05. Table 1 lists the various proteins and corresponding sequences where arginines were shown to be citrullinated. The LC MS/MS approach did not identify all the proteins that were detected through the use of the 6B3 anti-citrullinated protein antibody as containing citrullinated residues. Some possible explanations for this include, but are not limited to (1) overlapping of ion peaks due to similarity in mass with deamidation of Arg, (2) low abundance of citrullination modification to below the detection limit of the LC MS/MS technique but not western blot, (3) poor ionization of a citrullinated peptide, (4) poor fragmentation of a citrullinated peptide, and (5) non-specificity detection of the proteins by western blot.

**Table 1:** **Proteins identified to be citrullinated in response to TBI**.

Protein	Sequence	Mascot score	Function
Alpha-actinin-1	K.LRKDDPLTNLNT AFDVAER.Y	46.99	F-actin cross-linking protein which is thought to anchor actin to a variety of intracellular structures. http://www.uniprot.org/uniprot/Q9Z1P2
Amphiphysin	R.LQRELR.G	16.38	Involved in regulation of synaptic vesicle endocytosis and tumor suppression inhibiting malignant cell transformation. http://www.uniprot.org/uniprot/O08839
Creatine kinase B-type	K.LLIEMEQRLEQG QPIDDLMPAQK.	42.46	Creatine kinase isoenzymes play a central role in energy metabolism. http://www.uniprot.org/uniprot/P07335
Drebrin	R.LKDQSIFGDQRD EEEESQMK.K K.KALDARLR.F	45.82 29.54	Drebrins are thought to function in cell migration, extension of neuronal processes and plasticity of dendrites. http://www.uniprot.org/uniprot/Q16643
Dynamin-1	R.SGQASPSRPESPR PPFDL.	17.90	Mediates clathrin-endocytosis and other vesicular trafficking processes. http://www.uniprot.org/uniprot/A0A0A0MY51
Fructose-bisphosphate aldolase A	K.RALANSLACQGK.Y	10.24	Functions in glycolysis and gluconeogenesis. http://www.uniprot.org/uniprot/P05065
Heat shock cognate 71 kDa	K.VEIIANDQGNRTT PSYVAFTDTER.L	43.26	Acts as a repressor of transcriptional activation. Chaperone. http://www.uniprot.org/uniprot/P63018
Moesin	K.ERQEAEEAKEAL LQASR.D	21.64	Connects cytoskeletons to membranes. http://www.uniprot.org/uniprot/P26038
Myelin basic protein	R.FFSGDRGAPK.R K.GRGLSLSR.F	60.90 11.7	A highly abundant protein in the myelin membrane that surrounds neurons. http://www.uniprot.org/uniprot/P02688
NADH-ubiquinone oxidoreductase 75kDa subunit	R.IASQVAALDLGY KPGVEAIRK.N K.VALIGSPVDLTY RYDHLGDSPK.I	26.71 24.23	Core subunit of the mitochondrial membrane respiratory chain NADH dehydrogenase (Complex I). http://www.uniprot.org/uniprot/Q91VD9
Septin-9	R.RVETPASKAPEG SAMPVTDAAPK.R	13.24	Filament-forming cytoskeletal GTPase that is thought to play a role in cytokinesis. http://www.uniprot.org/uniprot/Q9QZR6
Synapsin-1	R.PVAGGPGAPPAA RPPASPSPQR.Q	38.25	A phosphoprotein of synaptic vesicles, thought to function in the regulation of neurotransmitter release. http://www.uniprot.org/uniprot/P09951
Synapsin-2	K.YDIRVQK.I	19.95	A phosphoprotein of synaptic vesicles, thought to function in the regulation of neurotransmitter release. May play a specific role in noradrenaline secretion by sympathetic neurons. http://www.uniprot.org/uniprot/Q63537

*Analysis of the mass spectra data set with a variable modification search for the citrullination of arginine residues revealed many sites of citrullination on arginine residues with Mascot score corresponding to *p* < 0.05. The LC MS/MS approach did not identify all the proteins that were detected through the use of the 6B3 anti-citrullinated protein antibody as containing citrullinated residues. Some possible explanations for this include, but are not limited to (1) overlapping of ion peaks due to similarity in mass with deamidation of Arg, (2) low abundance of citrullination modification to below the detection limit of the LC MS/MS technique but not western blot, (3) poor ionization of a citrullinated peptide, (4) poor fragmentation of a citrullinated peptide, and (5) non-specificity detection of the proteins by western blot*.

*Significance of red font: Arginine (R)*.

**Figure 9:**
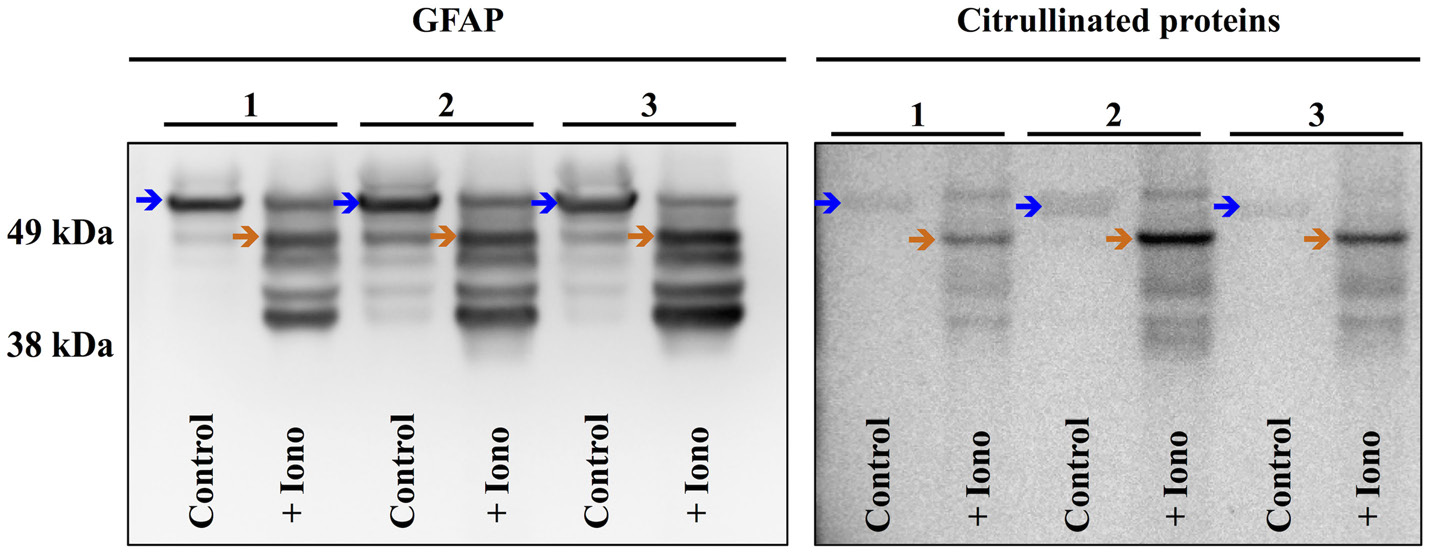
**Simulated brain injury in normal human astrocytes reveals a spectrum of GFAP breakdown products and the hyper-citrullination of one GFAP species**. Normal human astrocytes were treated with ionomycin (10 μM; 4 h) and analyzed for GFAP (left panel) and protein-bound citrulline immunoreactivity (right panel) by western blot. The results of three independent experiments (A–C) are presented, showing the immunoreactivity in extracts prepared from untreated control cells (Control) and cells treated with ionomycin (+Iono). The blue arrows indicate intact GFAP, while the orange arrows indicate the hyper-citrullinated GFAP breakdown product.

To further investigate the cellular mechanisms of injury-induced protein citrullination, a model of simulated TBI was established. In this model, normal human astrocytes (obtained commercially) were incubated in control medium or in medium containing the calcium ionophore, ionomycin, to induce calcium excitotoxicity. Figure 9 shows the results of three separate experiments investigating the effects of ionomycin treatment on the proteolytic processing of GFAP (left panel) and the generation of citrullinated proteins (right panel). The data show that treatment with ionomycin consistently activated the proteolytic processing of intact GFAP (left panel; blue arrows) to produce a distinctive pattern of breakdown products. Probing with mAb 6B3 indicated that one of the GFAP breakdown products is preferentially citrullinated (right panel; orange arrow) in response to simulated TBI. In addition, several other protein features appeared to be heavily citrullinated in response to ionomycin treatment. The identity of these signals remains to be determined.

## Discussion

Protein citrullination is a calcium-dependent protein modification that has been largely studied in the context of autoimmune disorders, particularly rheumatoid arthritis. Abnormal protein citrullination in rheumatoid arthritis results in the generation of antigenic epitopes that become targeted by the adaptive immune system (16, 24). Protein citrullination is also found to be abnormal in several neurodegenerative disorders, including MS and Alzheimer’s disease, suggesting that citrullinated proteins may also serve as antigenic targets in these conditions, and thereby contribute to long-term pathogenesis. To date, however, little research has examined the effects of TBI on protein citrullination, where an acute mechanical injury can lead to progressive and sustained neuropathology. Currently, there are no data regarding the identity of specific proteins affected by citrullination following TBI, or information on the susceptibility of different brain regions or cell types to this modification following injury.

The constellation of dysfunctions resulting from TBI is often complex and dynamic, involving pathology caused by acute tissue damage and interactions amongst the ongoing changes caused by ischemia, hypoxia, excitotoxicity, and elevated intracranial pressure (1). A hallmark of acute TBI pathophysiology, however, is intracellular calcium overload (25). This shift in ionic balance is excitotoxic, activating a number of damaging intracellular cascades, including lipid peroxidation, proteolysis, free radical generation (2), and the activation of pro-apoptotic genes (1) with the loss of dendritic spines. Elevated intracellular calcium is an essential condition for the activation of PAD enzymes, which catalyze the conversion of intrapeptidyl arginine residues to citrulline residues (26).

PAD enzymes exist as several isoforms, two of which are found in neural tissue: PAD2 (20, 27, 28) and PAD4 (29). In humans, PAD2 is largely localized to astrocytes, while PAD4 is exclusively expressed in neurons (29). Moreover, PAD2 is localized to reactive astrocytes in the brains of mice affected by a prion disorder, scrapie, where its enzymatic activity is nearly double that of healthy mice (20). Research on the etiology of MS has suggested that PAD2 contributes to the pathology of demyelination, where its activity is thought to destabilize myelin sheath structure (17).

The present investigation in rats indicates that TBI-induced protein citrullination is selectively localized to astrocytes. This finding is consistent with the understanding that following ischemia, astrocytes exhibit a dramatic rise in intracellular calcium due to a rapid influx of extracellular calcium through dysfunctional membrane channels as well as the release of calcium from intracellular stores (25). While influxes in intracellular calcium are also found in neurons following mechanical injury, this rise is far less profound than that observed in astrocytes (30). Furthermore, astrocytes selectively display long-term defects in calcium signaling following TBI (30) and ultimately, astrocytic death precedes that of neurons in rat models of TBI (25). Related findings involving hypoxia confirm that astroglial injury is due to massive calcium influxes and resulting excitotoxicity (26). Models of TBI illuminate the biologic importance of astrocytes in brain injury, serving a protective role by shielding neurons from damage due to oxygen deprivation (31), glutamate neurotoxicity, and calcium excitotoxicity (32). It has been proposed that the intracellular rise of calcium within astrocytes is directly linked to their neuroprotective effects on neurons, most likely due to their ability to sequester large amounts of calcium, and thus stabilize the ionic environment for neurons (33). Our finding that protein citrullination is limited to astrocytes is consistent with this proposal.

Present results indicate that the up-regulation of protein citrullination induced by TBI occurs in astrocytes that are located in the cerebral cortex, external capsule, and hippocampus. In contrast, the status of protein citrullination in other brain regions (such as the amygdala and caudate putamen) and cell types was not appreciably altered by TBI. The mechanistic basis for this observation may relate to the up-regulation of voltage-gated, class C L-type Ca^2+^ channels that are selectively expressed in astrocytes and are particularly sensitive to activation by injury in the regions reported here (34). The finding that nimodipine, an L-type calcium channel blocker, protects against excitotoxic damage in cultured astrocytes supports this proposal (35). These findings correspond to a model of calcium excitotoxicity-induced citrullination. The selective citrullination of astrocytes in these particular regions may correspond to long-term dysfunctions associated with TBI, including learning and memory deficits associated with progressive hippocampal atrophy (1). However, it is possible that citrullination may occur in other cell types at different time points following TBI, and future work is needed to address this possibility. Furthermore, a subset of astrocytes – in particular, perivascular astrocytes with end-feet contacting vessels, as well as the glia limitans underlying the pia mater – were particularly rich in citrullianted proteins. This may reflect a functional relevance of citrullinated proteins and their proximity to the blood–brain barrier, indicating a potential exposure of these modified molecules to the immune system, where citrullinated epitopes may stimulate the production of targeted immunogenicity.

Our investigation suggested that patterns of protein citrullination following TBI were similar between male and female rats. This finding is in contrast to our previous observations concerning the effects of TBI on protein carbonylation. Following injury, male rats showed far greater response in protein carbonylation as compared to female rats (18). Protein carbonylation is a reflection of oxidative stress (36), whereas citrullination is a marker of calcium influx (22, 37). Accordingly, the gender difference observed in carbonylation may be due to the protective antioxidant effects of ovarian steroids (38), whereas a similar mechanism does not appear to exist in the case of citrullination. The extent to which these protein modifications contribute to gender differences in TBI complications and mortality (39, 40) remains to be determined. It should be noted that the present experimental design focused on only one time point post-TBI (5 days), and as such, it is possible that gender differences in post-injury citrullination may be evident at other time points.

The present investigation has identified 37 proteins as a very small subset of the entire brain proteome that is possibly citrullinated in response to TBI. This potential selectivity in protein citrullination indicates that the mechanism involved maintains a high degree of specificity. Furthermore, several of the proteins identified here are reported to be citrullinated in neurodegenerative diseases, and thus may play causative roles in neuropathology. Specifically, citrullinated GFAP is a characteristic feature in MS and Alzheimer’s disease (11, 16, 41, 42), and MBP, a major component of myelin sheath structure, is profoundly over-citrullinated in MS (42, 43). Similarly, GFAP, tubulin, peroxiredoxin 1, cofilin-1, and alpha/gamma enolase are selectively citrullinated in prion disease (20). Therefore, the link between abnormal protein citrullination and neurological disease appears strong. It should be noted that the 37 proteins identified as targets for TBI-induced citrullination were derived from the analysis of only 16 gel features. Accordingly, not all of the proteins identified in a single feature are necessarily citrullinated, and may have resulted from the analysis due to their co-purification with another citrullinated species. Nevertheless, the high correlation that exists between the specific proteins potentially identified here and their reported citrullination in various disease states is consistent with the proposal that these proteins are indeed targets for citrullination following TBI.

A possible mechanism by which abnormal protein citrullination contributes to neurological disease could involve the adaptive immune system. A significant proportion of the proteins identified here are also recognized as autoantigens, in both neurological and autoimmune-related disorders. For example, MBP is the signature citrullinated autoantigen of MS (43, 44). Autoantibodies targeting amphiphysin are associated with several neurological disorders, including sensory neuronopathy and encephalopathy (45). Additionally, dihydropyrimidinase-related protein 2 (CRMP2) is autoantigenic in autoimmune retinopathy (46). Finally, alpha enolase has been identified as a citrullinated autoantigen in rheumatoid arthritis (22), while citrullinated 78 kDa glucose-regulated protein is an autoantigen within the pancreatic beta-cells in Type 1 diabetes (23). Collectively, these findings support the proposal that injury-induced protein citrullination may generate immunological epitopes that become targets of the adaptive immune system. This process may serve as an underlying basis for chronic and progressive neurological disorders.

Finally, this report presents the development of an *in vitro* model for simulating TBI in a controlled and cell-specific manner. Shown here in normal human astrocytes are the effects of calcium excitotoxicity, a hallmark condition of TBI, on the hyper-citrullination of GFAP. Moreover, the findings confirm the proteolytic processing of GFAP to a series of breakdown products that are consistent with those reported by others using *in vitro* models of TBI (47, 48). Interestingly, our investigation showed that one of these breakdown products is heavily citrullinated, and thus may serve as the antigen for the development of the anti-GFAP autoantibodies recently reported in TBI (49). Accordingly, the application to this and other models for simulated TBI may provide novel insights into consequences and mechanisms of TBI, and also identify informative biomarkers for assessing brain injury.

In conclusion, the present findings indicate that TBI may dramatically up-regulate protein citrullination within astrocytes in specific brain regions. Additionally, this modification may affect only a subset of the neural proteome, primarily affecting proteins involved in cytoskeletal structure, metabolic processes, and cell–cell signaling. A large proportion of these proteins have been identified as citrullinated in other pathologies, including MS, Alzheimer’s disease, and rheumatoid arthritis, indicating a potential role for this protein modification in ongoing pathological processes. Interestingly, gender does not affect the degree or distribution of this modification in neural tissue, in contrast to previous observations involving TBI-induced protein carbonylation. Accordingly, gender differences in the CNS response to TBI may not involve differential responses in protein citrullination. Finally, the work presented here examined protein citrullination only at 5 days following injury. According future work is needed to address the presence of citrullination following TBI in an extended time course to investigate the long-lasting effects of TBI on the status of protein citrullination in the central nervous system. In summary, this research indicates that abnormal protein citrullination is a possible feature of TBI that may contribute to ongoing pathological mechanisms following acute injury, potentially including aspects of autoimmune dysfunction.

## Author Contributions

All authors were involved in drafting the manuscript or revising it critically for important intellectual content to include the final approval of this version for publication. RL and GP Mueller substantially contributed to the conception and design of the work, analysis and interpretation of the data. JB, MF, JF, GH, GP Martinelli, and DJ substantially contributed to the acquisition of the data. All agree to be accountable for all aspects of the work in ensuring that questions related to the accuracy or integrity of any part of the work are appropriately investigated and resolved.

## Conflict of Interest Statement

The authors declare that the research was conducted in the absence of any commercial or financial relationships that could be construed as a potential conflict of interest.

## Supplementary Material

The Supplementary Material for this article can be found online at http://journal.frontiersin.org/article/10.3389/fneur.2015.00204
