# Supplementary material for: Protein Citrullination: A Proposed Mechanism for Pathology in Traumatic Brain Injury
Source: Front Neurol. 2015 Sep 22;6:204. doi: 10.3389/fneur.2015.00204 (PMC4585288; doi:10.3389/fneur.2015.00204)
Supplement: Supplementary file 1 [file Presentation_1.PDF]

Supplementary Material

Protein citrullination: A proposed mechanism for pathology in traumatic brain injury

Rachel C. Lazarus[1], John E. Buonora[2], Michael N. Flora[3], James G. Freedy[3], Gay R. Holstein[4], Giorgio P. Martinelli[4], David M. Jacobowitz[1,3], and Gregory P. Mueller[1,3,5]*

*** Correspondence:** Gregory P. Mueller, USUHS, C2117, 4301 Jones Bridge Rd, Bethesda, MD, USA 20814-4799
gregory.mueller@usuhs.edu

1.1. Supplementary Figures

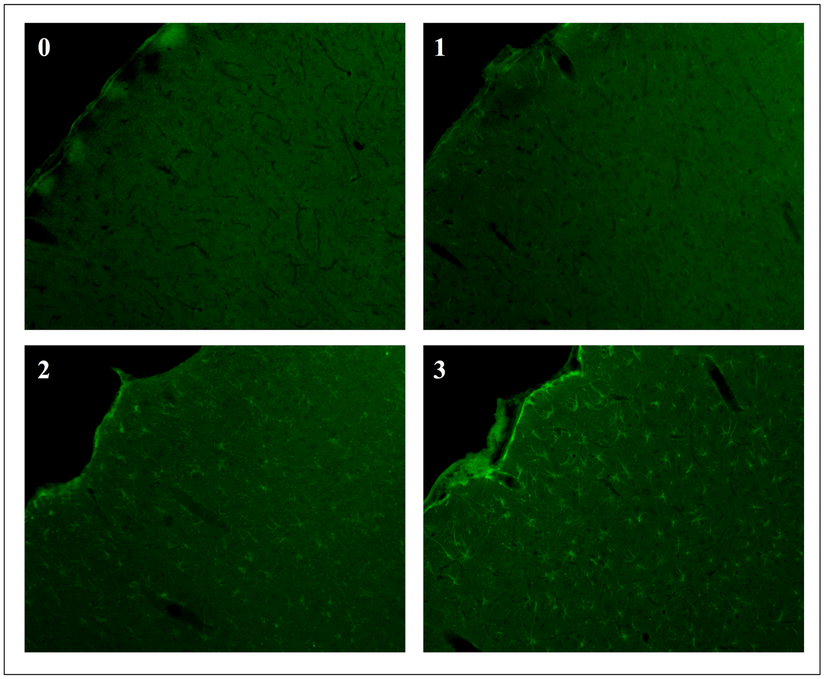

Supplementary Figure 1. Representative images of the citrullination scoring system in the cortex. Brain tissue collected five days following CCI was evaluated with mAb 6B3 for the presence of citrullinated proteins. Images show relative increases in citrullination immunofluorescence (scored at 0-3) in the cortex, original magnification x10.

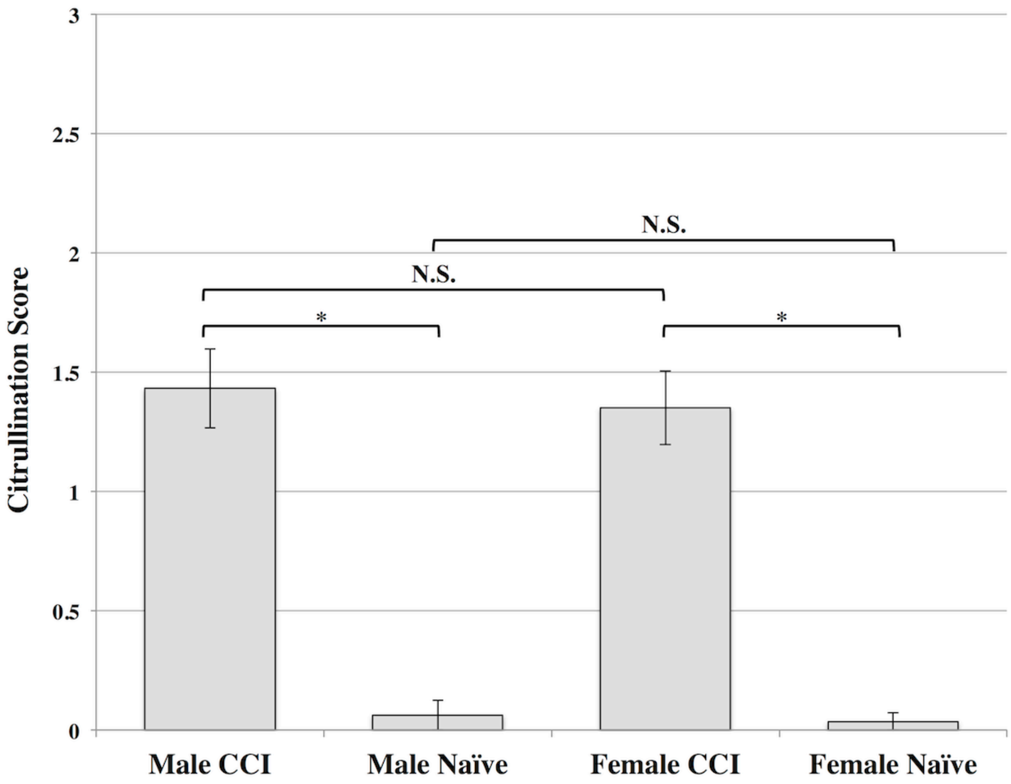

Supplementary Figure 2. Effects of CCI on protein citrullination in the cortex of male and female rats. CCI produced a marked increase in protein citrullination throughout the injured cortex, extending from lateral to the lesion site to regions of the cortex not directly impacted by CCI. Scoring of this region revealed that male naïve rats (mean score: 0.06) and male CCI rats (mean score: 1.43) were significantly different (*), $p < 0.001$, and female naïve rats (mean score: 0.04) and female CCI rats (mean score: 1.35) were also significantly different (*), $p < 0.001$ (n = 11 male rats, CCI; n = 8 male rats, naïve control; n = 10 female rats, CCI; n = 7 female rats, naïve control) ($F(3,32)=31.05$, $p = 0.000$, with post hoc Tukey HSD analyses considering Injury and Gender variables independently). Data indicate means ± SEM. N.S.: Not statistically significant difference.
